# Compact Ultra-Wideband Wilkinson Power Divider in Parallel Stripline with Modified Isolation Branches

**DOI:** 10.3390/s24113437

**Published:** 2024-05-27

**Authors:** Dong-Jae Go, Byung-Cheol Min, Mun-Ju Kim, Hyun-Chul Choi, Kang-Wook Kim

**Affiliations:** School of Electronic and Electrical Engineering, Kyungpook National University, Daegu 41566, Republic of Korea; dongjae.go1@gmail.com (D.-J.G.); minbc4658@knu.ac.kr (B.-C.M.); dranswn@knu.ac.kr (M.-J.K.); hcchoi@ee.knu.ac.kr (H.-C.C.)

**Keywords:** parallel stripline, power divider/combiner, ultra-wideband, isolation branch, optimization algorithm

## Abstract

An efficient design method for a compact and ultra-wideband multi-stage Wilkinson power divider in a parallel stripline (PSL) is proposed. To enhance the frequency bandwidth of the proposed power divider while reducing its size, the isolation branch is modified; that is, two capacitors are connected to both sides of a resistor at each isolation branch. For an efficient design process, the PSL power divider is equivalently represented by two microstrip power dividers, and the design equations are derived. Based on the design equations, an in-house algorithm is utilized to optimally determine the design parameters, including the line impedance, resistance, and capacitance of each stage. For example, a three-stage PSL power divider is designed with three λ/4 transmission lines at a base frequency of 5 GHz. To verify the accuracy of the design procedure, 3D EM simulations and measurements are performed, and the results show good agreement. Compared with the conventional three-stage Wilkinson power divider, the proposed PSL power divider achieves a wider frequency bandwidth of 1.16 to 6.51 GHz (139.5%) and a 23% shorter transmission line length of 207°, while exhibiting an insertion loss of 0.7 to 1.4 dB.

## 1. Introduction

As 5G and beyond-5G technologies such as artificial intelligence and autonomous driving are further developed, there is a strong demand for higher-speed digital data transmission. For this purpose, recently, various studies on wideband transmission lines and components for very high-speed digital signals have been actively performed [1,2,3,4]. The conventional transmission line used for high-speed digital transmission is a differential line (DL). The DL consists of two parallel microstrip lines (MSLs) with opposite polarities, supports differential signaling, and has two signal lines and a ground plane. However, as the transmission speed of the digital signals increases, significant issues may arise due to a signal skew caused by a length mismatch between the two signal lines of a DL or an unequal electromagnetic wave interference. These problems can result in phase imbalances, signal integrity issues, and malfunctions in receiving devices [4,5,6].

For the ultra-high-speed digital transmission in typical PCBs, ultra-wideband transitions from a DL to a balanced line (BL), such as a coplanar stripline (CPS) or a parallel stripline (PSL), were proposed [4]. The frequency bandwidth of the BL-based transmission lines can be more than four times wider than that of the DL-based transmission lines, enabling a significant increase in the digital transmission speed. These BL-based transmission lines provide effective noise rejection, common-mode signal reduction, and autonomous phase-recovery properties [4]. A PSL is a transmission line with only two signal lines, that is, one on the top layer and the other on the bottom layer. Each signal line acts as a ground reference for the other, resulting in strong electromagnetic coupling between the two lines. Therefore, a high-performance PSL power divider with an ultra-wide bandwidth will be very useful in dividing or combining wideband balanced signals.

To design wideband power dividers, many techniques have been developed, such as using coupled lines [7,8], a ring structure [9], and artificial transmission lines implemented with lumped elements [10]. A popular design approach for wideband Wilkinson power dividers using the multi-stage topology was suggested by Cohn [11]. In [11], for the multi-stage topology, each transmission line section had a different characteristic line impedance along with an isolation branch with a different resistor value. Many research efforts have been carried out to develop wideband power dividers on the basis of Cohn’s multi-stage topology, using various planar transmission lines such as an MSL [12], stripline [13], and PSL [14]. However, the size of these conventional multi-stage Wilkinson power dividers increases by a step of a λ/4 transmission line for each additional stage. In order to reduce the overall size while retaining or improving the performance, several researchers modified the resistive isolation branches and successfully implemented compact multi-stage power dividers [15,16]. In [15], with a center frequency of 2 GHz, small capacitors as low as ~0.02 pF were connected to the resistors in the isolation branch. As the operating frequency bandwidth increased, however, the required capacitance values became smaller and impractical to be implemented. In [16], the size of a multi-stage power divider was effectively reduced by connecting series and shunt capacitors to the isolation branch and by employing a defected ground structure (DGS). The implementation of this power divider, however, could encounter a difficulty since the required line impedance increases significantly as the number of stages increases.

On the other hand, research efforts have been made to implement PSL wideband power dividers. In [17], a three-stage wideband PSL power divider was proposed by optimizing the transmission lines of a dual-band power divider, but due to using fixed resistor values, the isolation level between the output ports was not high. As another approach for a wideband PSL power divider, a ring power divider was proposed [18], which employed a 180° phase shifter to achieve high isolation performance. However, with that configuration of the PSL divider, it was difficult to obtain a broader bandwidth than that of the conventional multi-stage topology.

In this paper, an efficient design approach to implement a compact and ultra-wideband PSL Wilkinson power divider is proposed. In the proposed design, to expand the bandwidth of the power divider while reducing the size, the isolation branch at each stage of the multi-stage power divider is modified by adding two capacitors to both sides of the resistor. Also, the proposed PSL power divider is modeled as two MSL power dividers, as discussed in [19], and the design equations are derived by using even–odd mode analysis and ABCD matrices. Furthermore, an in-house optimization algorithm is utilized to determine the optimal design parameters, based on the design equations, without performing EM simulations. To verify the design procedure and provide a design example, a three-stage PSL power divider is designed, fabricated, and measured. The proposed power divider achieves a wider bandwidth and a smaller size compared to the conventional designs. Moreover, it is implemented without requiring extremely low capacitance or high impedance values.

## 2. Design of a Three-Stage Power Divider in a Parallel Stripline

### 2.1. Configuration

**Figure 1:**
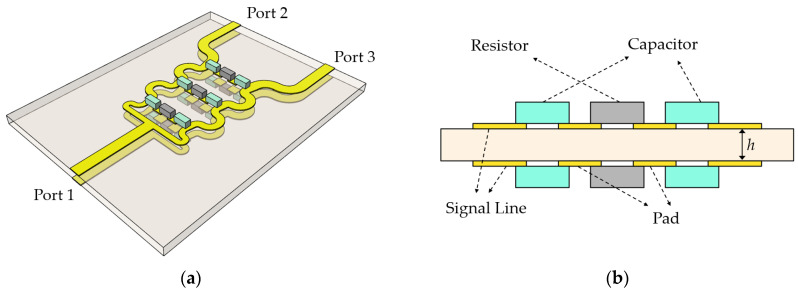
(**a**) Perspective view of the proposed power divider, and (**b**) cross-sectional view of the isolation branch at each stage.

In Figure 1a, a perspective view of the proposed three-stage PSL power divider is shown, and an enlarged cross-section of an isolation branch at each stage is illustrated in Figure 1b. On the circuit substrate, the upper and lower structures and components are laid out symmetrically with respect to the midplane. Also, on the upper and lower layers, a capacitor is connected to each side of the isolation resistor, respectively, with an additional pad stub used to place the capacitor and resistor. The design parameters of the proposed power divider are the values of the line impedance, resistance, and capacitance at each stage.

**Figure 2:**
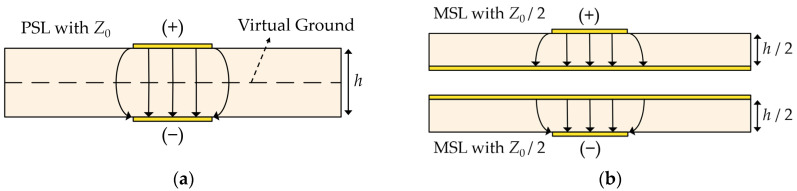
(**a**) Cross-sectional view of the PSL with the characteristic impedance of $Z_{0}$, and (**b**) equivalent structures consisting of two MSLs with $Z_{0}/2$.

The proposed PSL-based structure is symmetric with respect to the midplane of the substrate and can be analyzed using the image theory [19]. Figure 2a illustrates the cross-section of the PSL with the characteristic impedance of $Z_{0}$ and the simplified electric field lines. As can be seen, the upper and lower signal lines possess the opposite polarities, and on the midplane of the substrate, a virtual ground plane can be placed. Therefore, the PSL is equivalent to two identical MSLs with the characteristic impedance of $Z_{0}/2$ and half the substrate thickness (h/2), as shown in Figure 2b. Since the two MSLs in Figure 2b have symmetric structures with the opposite polarities, the rest of the design process is performed with one of the MSL structures.

### 2.2. Analysis

**Figure 3:**
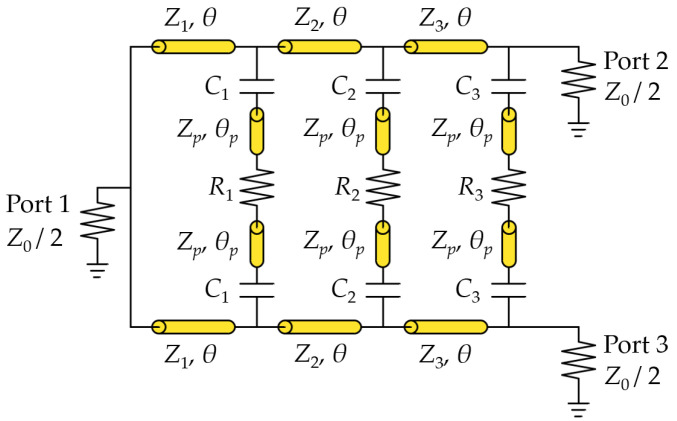
Equivalent circuit for an MSL-converted structure of the proposed power divider.

Figure 3 illustrates an equivalent circuit for one of the MSL-converted structures of the proposed PSL power divider. Each transmission line section has a characteristic impedance of $Z_{1}$, $Z_{2}$, and $Z_{3}$, respectively, with the same electrical length θ, which is the quarter-wave length (θ = 90°) at the base frequency $f_{0}$. With a conventional multi-stage Wilkinson power divider, the base frequency $f_{0}$ typically becomes the center frequency of the power divider. By including the capacitors in the isolation branch, however, the center frequency typically shifts to a lower value than $f_{0}$. Therefore, in the proposed design, the base frequency $f_{0}$ is selected as a reference frequency for design convenience. In the figure, the values of the resistors in the isolation branch are $R_{1}$, $R_{2}$, and $R_{3}$, and the values of capacitors are $C_{1}$, $C_{2}$, and $C_{3}$, respectively. Also, $Z_{p}$ and $\theta_{p}$ are the pad stub parameters.

**Figure 4:**
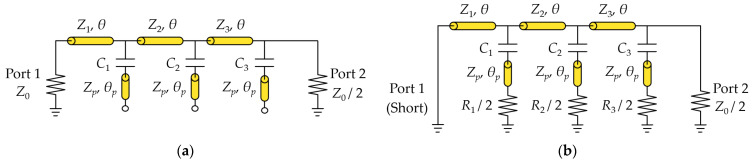
Bisected even- and odd-mode circuits: (**a**) even-mode, and (**b**) odd-mode.

The circuit in Figure 3 can be analyzed by considering the even- and odd-mode configurations since Ports 2 and 3 are symmetric with respect to Port 1. If positive voltage sources are simultaneously applied to Ports 2 and 3, no current will flow through the isolation resistors ($R_{1}$, $R_{2}$, $R_{3}$), and virtual open-terminations can be placed at the middle points of the circuit. In this case, an equivalent even-mode circuit can be formed, as shown in Figure 4a, where each shunt element consists of a capacitor and an open-terminated stub. Similarly, if voltage sources with opposite polarities are applied to Ports 2 and 3, respectively, the virtual grounds can be placed at the middle points of the circuit, and an equivalent odd-mode circuit is shown in Figure 4b.

The equivalent even- and odd-mode circuits in Figure 4a,b can be analyzed by calculating the ABCD matrices and converting them into the corresponding S-parameters. The ABCD matrix for the transmission line section for each stage is given in (1). Also, the ABCD matrix for the shunted elements of the even-mode circuit for each stage is represented by (2), and the ABCD matrix for the shunted elements of the odd-mode circuit for each stage is given in (3).

(1) $${\left[\begin{array}{cc} A & B\\ C & D \end{array}\right]}_{t,i}=\left[\begin{array}{cc} \;\cos\theta & jZ_{i}\sin\theta \\ j\sin\theta /Z_{i} & \cos\theta \end{array}\right]$$

(2) $${\left[\begin{array}{cc} A & B\\ C & D \end{array}\right]}_{e,i}=\left[\begin{array}{cc} 1 & 0\;\;\;\\ \frac{1}{\frac{1}{j\omega C_{i}}-jZ_{p}\cot\theta_{p}} & 1\;\;\; \end{array}\right]$$

(3) $${\left[\begin{array}{cc} A & B\\ C & D \end{array}\right]}_{o,i}=\left[\begin{array}{cc} 1 & 0\;\\ \frac{1}{\frac{1}{j\omega C_{i}}+Z_{p}\frac{(R_{i}/2)+jZ_{p}\tan\theta_{p}}{Z_{p}+j(R_{i}/2)\tan\theta_{p}}} & 1\; \end{array}\right]$$

where i = 1, 2, 3 (stage number). When a signal is applied at Port 1, the total ABCD matrix can be calculated by sequentially multiplying the corresponding ABCD matrices, as shown in (4). Here, the subscripts (*m*,*i*) represent each even-mode (*e*,*i*) or odd-mode (*o*,*i*) shunt circuit for the stage number *i*. For the even-mode circuit, the matrices in (1) and (2) with the corresponding parameters are sequentially multiplied, and also for the odd-mode circuit, the matrices in (1) and (3) with the corresponding parameters are multiplied. When a signal is applied at Port 2, the matrices are multiplied in the reverse order, as given in (5).

(4) $${\left[\begin{array}{cc} A & B\\ C & D \end{array}\right]}_{m,p1}={\left[\begin{array}{cc} A & B\\ C & D \end{array}\right]}_{t,1}{\left[\begin{array}{cc} A & B\\ C & D \end{array}\right]}_{m,1}{\left[\begin{array}{cc} A & B\\ C & D \end{array}\right]}_{t,2}{\left[\begin{array}{cc} A & B\\ C & D \end{array}\right]}_{m,2}{\left[\begin{array}{cc} A & B\\ C & D \end{array}\right]}_{t,3}{\left[\begin{array}{cc} A & B\\ C & D \end{array}\right]}_{m,3}$$

(5) $${\left[\begin{array}{cc} A & B\\ C & D \end{array}\right]}_{m,p2}={\left[\begin{array}{cc} A & B\\ C & D \end{array}\right]}_{m,3}{\left[\begin{array}{cc} A & B\\ C & D \end{array}\right]}_{t,3}{\left[\begin{array}{cc} A & B\\ C & D \end{array}\right]}_{m,2}{\left[\begin{array}{cc} A & B\\ C & D \end{array}\right]}_{t,2}{\left[\begin{array}{cc} A & B\\ C & D \end{array}\right]}_{m,1}{\left[\begin{array}{cc} A & B\\ C & D \end{array}\right]}_{t,1}$$

The reflection coefficient in a two-port network with asymmetric characteristic impedances can be obtained with (6), using the ABCD parameters from (4) or (5).

(6) $$\Gamma =\frac{AZ_{L}+B-CZ_{S}Z_{L}-DZ_{S}}{AZ_{L}+B+CZ_{S}Z_{L}+DZ_{S}}$$

where $Z_{S}$ and $Z_{L}$ are the input and output impedances, respectively. As shown in Figure 4, with the input of Port 1, $Z_{L}=Z_{0}/2$ and $Z_{S}=Z_{0}$ (even mode) or $Z_{S}=0$ (odd mode). Similarly, with the input of Port 2, $Z_{S}=Z_{0}/2$, and $Z_{L}=Z_{0}$ (even mode) or $Z_{L}=0$ (odd mode).

Therefore, the return loss ($S_{11}$) and isolation ($S_{32}$) of the proposed power divider can be obtained [16] as

(7) $$S_{11}=\Gamma_{e,p1}$$

(8) $$S_{32}=\frac{1}{2}\Gamma_{e,p2}-\frac{1}{2}\Gamma_{o,p2}$$

where $\Gamma_{e,p1}$ and $\Gamma_{e,p2}$ are the even-mode reflection coefficients with the Port 1 and Port 2 inputs, respectively, obtained by using (6) and the equivalent circuit in Figure 4a. In a similar manner, $\Gamma_{o,p2}$ is the odd-mode reflection coefficient with the Port 2 input and can be obtained using (6) and the equivalent circuit in Figure 4b. By adjusting the values of the resistors and capacitors, the bandwidth of the return loss and isolation can be maximized.

### 2.3. Optimization Algorithm

**Figure 5:**
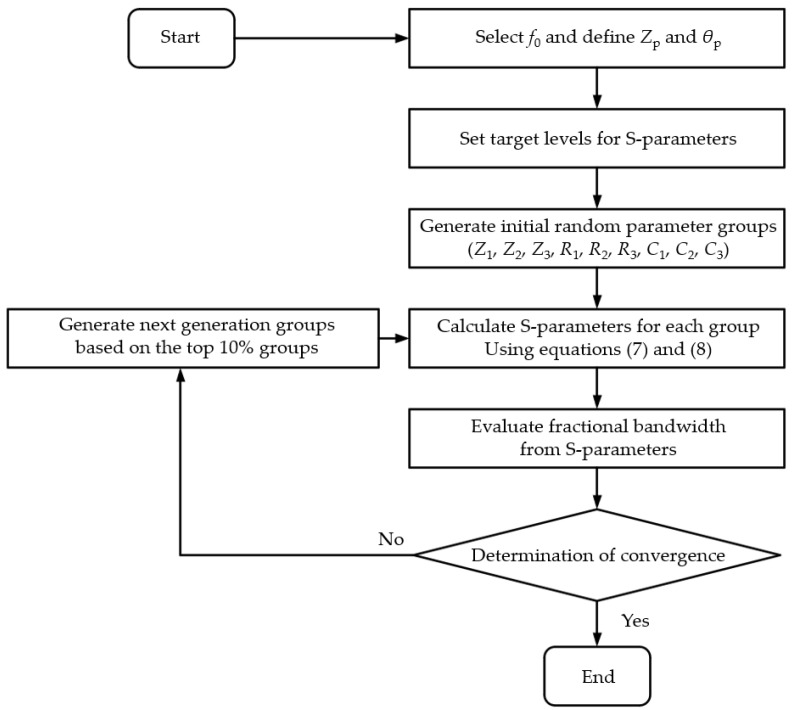
Flowchart of the optimization algorithm for designing the proposed power divider.

This section describes an in-house optimization algorithm used to efficiently determine the design parameters ($Z_{1}$, $Z_{2}$, $Z_{3}$, $R_{1}$, $R_{2}$, $R_{3}$, $C_{1}$, $C_{2}$, $C_{3}$) for the equivalent MSL circuit of the proposed power divider, as presented in Figure 3, with the optimization goal of maximizing the bandwidth. Figure 5 is a flowchart of the optimization algorithm for designing the proposed power divider.

The proposed algorithm, similarly to the genetic algorithm, starts by randomly generating the initial parameter groups. At each iteration, the performances of the circuit with the given parameter groups are compared with those of the other groups, and the parameter groups are updated to the next-generation groups showing better performances. The optimization procedure is summarized as follows: (1) A base frequency $f_{0}$ is selected according to the desired operation bandwidth of the power divider so that the length of the transmission line at each stage (θ) of the power divider is set as a quarter wavelength at $f_{0}$. (2) Also, the values of $Z_{p}$ and $\theta_{p}$, which correspond to the pad stub size, are initially chosen. (3) Then, 100 groups of design parameters are randomly generated. For an efficient design, appropriate initial parameters can be selected. The line impedances ($Z_{1}$, $Z_{2}$, and $Z_{3}$) and resistances ($R_{1}$, $R_{2}$, and $R_{3}$) can be chosen from the parameters of the conventional multi-stage power divider in [11], and the capacitance values ($C_{1}$, $C_{2}$, and $C_{3}$) can be chosen such that $X_{C}=Z_{0}/2$ (port impedance of one of the MSLs) at the base frequency $f_{0}$. (4) The S-parameters are calculated using (7) and (8) from 0 to 2$f_{0}$ with a step size of 0.01$f_{0}$. (5) The fractional bandwidth of each group is evaluated from the calculated S-parameters according to the target levels for the return loss and isolation, and they are compared with the other groups to select the top 10% performing groups. (6) Next-generation groups based on the top 10% groups are generated, and the process is iterated until there is no further improvement in the bandwidth. Through the proposed algorithm, it is possible to find the design parameters for the power divider required to produce an ultra-wide bandwidth with a center frequency lower than the base frequency $f_{0}$.

As an example of the proposed optimization algorithm, the base frequency is selected as $f_{0}=5$ GHz, and the characteristic impedance is selected as $Z_{0}=50$ Ω. The pad stub size is selected as 0.762 mm × 1.016 mm to fit with the lumped resistors and capacitors so that $Z_{p}=41.3$ Ω and $\theta_{p}=10$° at $f_{0}=5$ GHz. The target levels are set as a return loss of 20 dB and an isolation of 30 dB, where the target levels include an extra margin of at least 10 dB by considering the possible performance degradation due to fabrication tolerances in the actual implementation. To determine the convergence, it is assumed that the converged result is acquired when the difference of the evaluated fractional bandwidths (FBWs) is less than 0.5% during the most recent five iterations. The converged result is obtained with 26 iterations. Due to the randomness of this method, however, the iteration required for convergence may be changed for each run, even with the same configuration.

**Figure 6:**
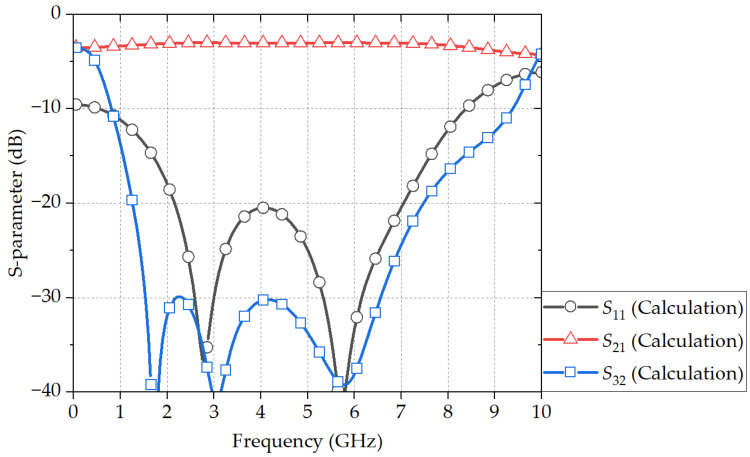
Calculated S-parameters of the equivalent circuit of the proposed power divider with the design parameters in Table 1.

Table 1 lists the obtained design parameters using the described optimization algorithm. Figure 6 shows the calculated S-parameters using the obtained design parameters. With a return loss of 10 dB and an isolation of 18 dB, without an extra margin, the center frequency is 4.48 GHz, which is $0.90f_{0}$, having the frequency bandwidth of 1.20 to 7.75 GHz (146.4%).

## 3. Simulations

**Figure 7:**
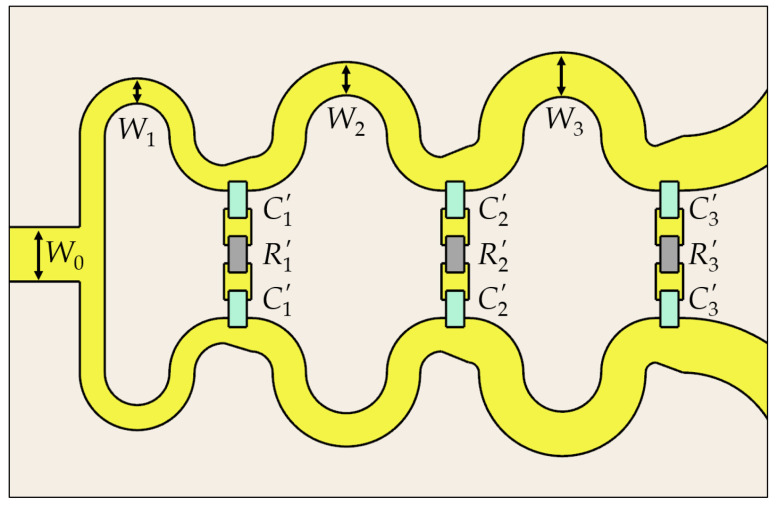
Top view of the proposed PSL power divider. The bottom view is the same.

The performance of the proposed PSL power divider with the design parameters obtained with the in-house optimization algorithm (Table 1) is verified using the commercial 3D EM simulator (CST Microwave Studio). Figure 7 illustrates a layout of the proposed PSL power divider, which is implemented with the Rogers RO4003C substrate ($\varepsilon_{r}=3.38$, tanδ=0.0027), with a thickness of h=0.508 mm. The design parameters in the physical dimension are listed in Table 2, where $W_{0}$ is the input/output port width, and $W_{1}$, $W_{2}$, $W_{3}$ are the widths of the line stages, respectively. $R_{1}^{\prime }$, $R_{2}^{\prime }$, $R_{3}^{\prime }$ and $C_{1}^{\prime }$, $C_{2}^{\prime }$, $C_{3}^{\prime }$ are the actual values of the resistors and capacitors used in the isolation branches, which are the commercial standard values close to the calculated ones.

**Figure 8:**
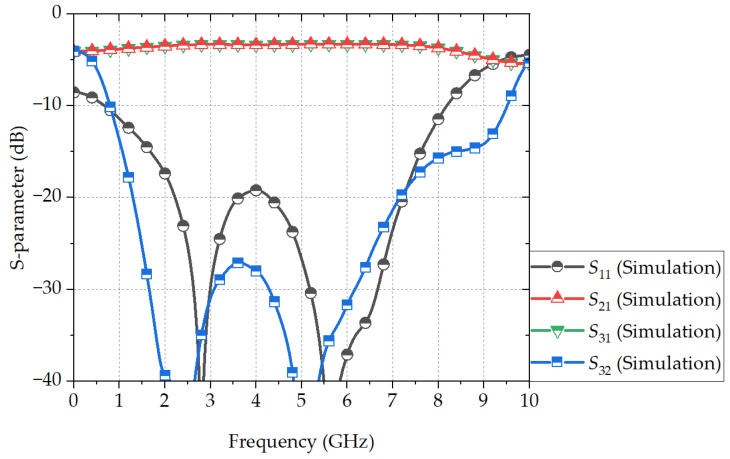
EM-simulated S-parameters of the proposed PSL power divider.

Figure 8 shows the EM-simulated S-parameters of the proposed PSL power divider. The frequency bandwidth with a return loss of 10 dB and an isolation of 18 dB is 1.21 to 7.46 GHz (144.2%). The center frequency is 4.34 GHz, which is $0.87f_{0}$ with the 234° electrical length. The difference in the frequency bandwidth between the calculation and the EM simulation is ~2.2%, proving the accuracy of the proposed design method. The insertion loss within the operation bandwidth is between 0.3 and 0.7 dB.

**Figure 9:**
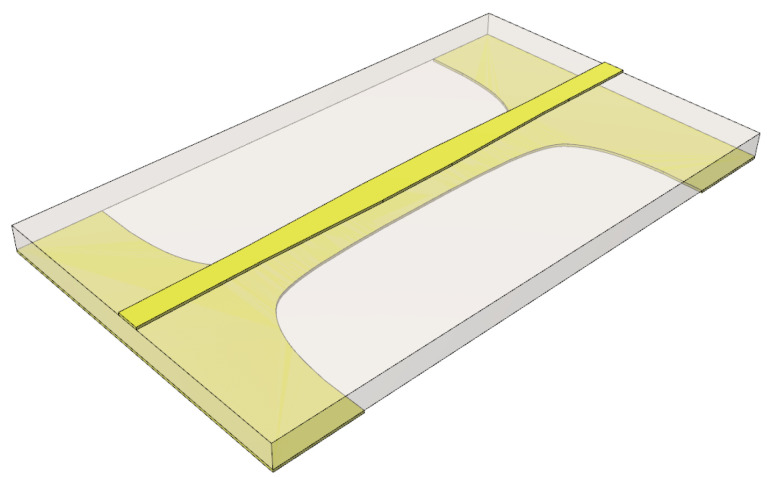
Perspective view of the MSL-to-PSL transition in a back-to-back configuration.

**Figure 10:**
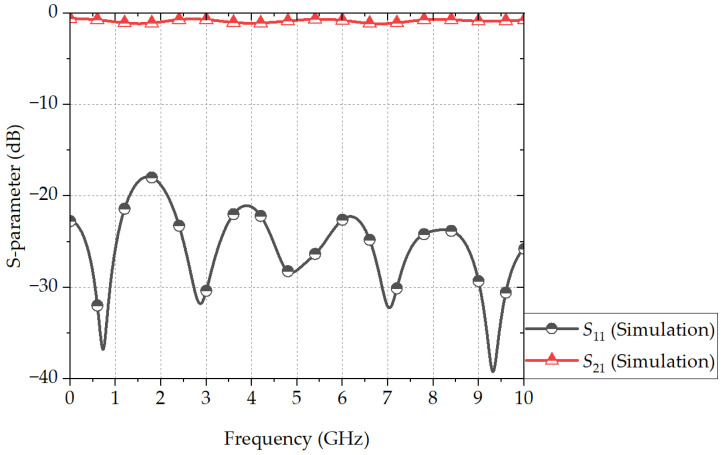
EM-simulated S-parameters of the back-to-back MSL-to-PSL transition.

The proposed PSL power divider has PSL input/output ports, and for the measurements with a commercial two-port vector network analyzer (VNA; Anritsu MS4644B), an MSL-to-PSL transition and an SMA end-launcher are connected to each input/output port of the PSL power divider. An ultra-wideband MSL-to-PSL transition was proposed by the authors’ group [20], and Figure 9 shows a perspective view of the 50 Ω MSL-to-PSL transition in a back-to-back configuration. Figure 10 shows the EM-simulated S-parameters of the MSL-to-PSL transition, and the insertion loss ranges between 0.3 and 0.6 dB per transition for the frequency bandwidth of near DC to 10 GHz.

## 4. Fabrication and Measurements

**Figure 11:**
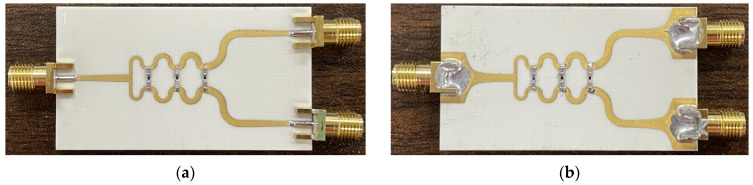
Pictures of the fabricated PSL power divider with the 50 Ω MSL-to-PSL transition: (**a**) top side, and (**b**) bottom side.

**Figure 12:**
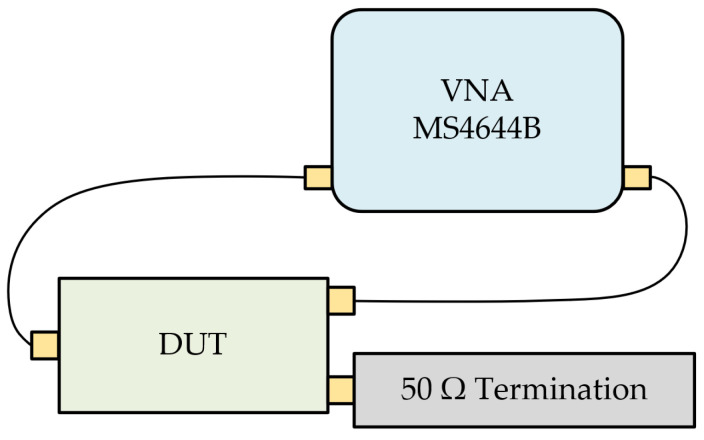
Simple diagram of the measurement setup.

The proposed PSL power divider is fabricated using the Rogers RO4003C substrate, with a thickness of h=0.508 mm. The MSL-to-PSL transitions are also implemented at the input/output ports for the measurements. Figure 11 shows the top and bottom sides of the fabricated power divider, and the size of the substrate is 55 mm × 31 mm. To measure the proposed power divider with a two-port VNA, a 50 Ω termination is connected at the remaining port. Figure 12 shows a simple diagram of the measurement setup.

**Figure 13:**
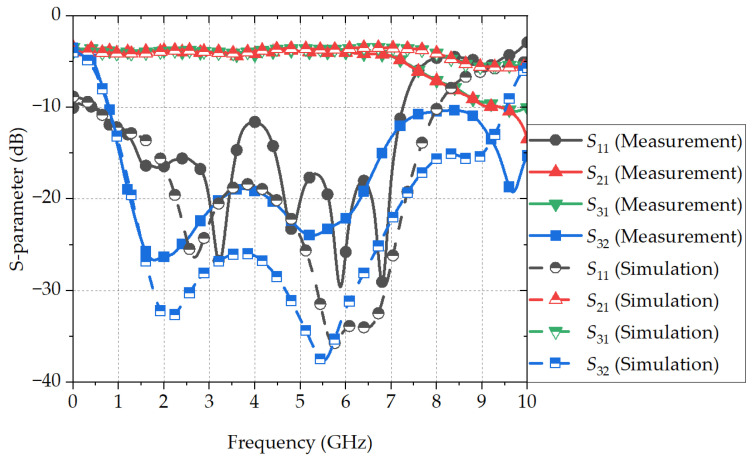
EM-simulated and measured S-parameters of the proposed PSL power divider connected with a 50 Ω MSL-to-PSL transition.

Figure 13 shows the measured S-parameters of the proposed PSL power divider, as compared with the simulated results. The measured bandwidth for a return loss of 10 dB and an isolation of 18 dB is observed from 1.16 to 6.51 GHz (139.5%), and the EM-simulated bandwidth ranges from 1.21 to 7.54 GHz (144.7%). These results agree well with the calculated S-parameter bandwidth of 1.20 to 7.75 GHz (146.4%). The center frequency is 3.84 GHz, which is $0.77f_{0}$, with the electrical length of 207°. The insertion loss within the operation bandwidth lies between 0.7 and 1.4 dB, which includes the insertion loss of the MSL-to-PSL transition and the SMA end-launcher. Also, the difference in the insertion loss between the output ports is less than 0.2 dB.

The performance of the proposed PSL power divider is compared with those of the reported wideband power dividers in Table 3. For comparison, Table 3 lists the performance of a conventional three-stage Wilkinson power divider using ideal transmission lines, designed according to the guideline in [11], with a center frequency of 3.84 GHz and a minimum isolation of 27.9 dB through a simulation with the commercial RF circuit simulator (Cadence AWR Microwave Office), resulting in a 126% fractional bandwidth. The measured fractional bandwidths of the reported three-stage power dividers are less than ~120%, while that of the proposed power divider is measured as 139.5%. Also, the electrical size of the proposed power divider (207°) is 23% more compact than that of the conventional three-stage Wilkinson power divider (270°).

## 5. Conclusions

This paper proposes an efficient design method for a compact and ultra-wideband Wilkinson power divider in a PSL. To widen the bandwidth of the multi-stage power divider while reducing the size, the isolation branch is modified by connecting two capacitors in series with a resistor at each stage. For the analysis, the PSL power divider is equivalently converted into two identical MSL power dividers. Using the derived design equations from the MSL power divider, the design parameters are obtained by utilizing a simple in-house optimization algorithm. Consequently, the performance of the proposed power divider can be efficiently optimized for the desired frequency bandwidth without performing 3D EM simulations. In order to validate the accuracy of the design procedure, EM simulations and measurements have been performed. As an example, a three-stage PSL power divider at a base frequency of 5 GHz is designed. The fabricated PSL power divider provides the frequency bandwidth of 1.16 to 6.51 GHz (139.5%), with an insertion loss between 0.7 and 1.4 dB. The proposed power divider can be designed without employing narrow transmission lines, via holes, and a DGS that may cause manufacturing complexities. The results of the calculations, EM simulations, and measurements agree very well. Also, the proposed design approach can be utilized for designing MSL power dividers, not only for PSL. The proposed compact PSL power divider can be applied for the ultra-wideband RF front-end and the differential signaling for high-speed digital data transmission.

## Figures and Tables

**Table 1:** Design parameters obtained by the optimization algorithm.

$Z_{1}$	$Z_{2}$	$Z_{3}$	$R_{1}$	$R_{2}$	$R_{3}$	$C_{1}$	$C_{2}$	$C_{3}$
43.7 Ω	36.0 Ω	29.2 Ω	43.1 Ω	114.9 Ω	184.6 Ω	3.34 pF	3.70 pF	1.71 pF

**Table 2:** Design parameters of the proposed PSL power divider.

$W_{0}$	$W_{1}$	$W_{2}$	$W_{3}$	$R_{1}^{\prime }$	$R_{2}^{\prime }$	$R_{3}^{\prime }$	$C_{1}^{\prime }$	$C_{2}^{\prime }$	$C_{3}^{\prime }$
1.52 mm	0.70 mm	0.93 mm	1.24 mm	43 Ω	110 Ω	180 Ω	3.3 pF	3.7 pF	1.7 pF

**Table 3:** Performance comparison between the proposed power divider and the reported power dividers.

Reference	Topology	Center Frequency (GHz)	Insertion Loss (dB)	Return Loss (dB)	Isolation (dB)	Fractional Bandwidth	Total Electrical Length (°) ^2^
[7]	MSL WPD * with coupled line	6.8	0.4 @ 6.8 GHz	10	10	97.1%	180
[8]	MSL WPD with coupled line	2.5	0.7	10	17.5	78%	270
[9]	MSL WPD with ring structure	3	0.8 (max)	10	10	100%	101.25
[10]	MSL WPD with artificial transmission line	0.125 0.3	-	17	18	100% 102.5%	-
[11]	Three-stage conventional WPD ^1^	3.84	0.1 (max)	18	18	126%	270
[15]	Three-stage MSL WPD with parallel RC isolation branches	2	0.2	15	15	117%	179
[16]	Three-stage MSL WPD with RC isolation branches and DGS	0.5	0.47 @ 0.5 GHz	18	18	108%	90
[17]	Three-stage PSL WPD	4	1.3 (max)	10	10	100%	-
[18]	PSL ring power divider	2	0.7 (max)	10	25	96.5%	180
This work	Three-stage PSL WPD with series RC isolation branches	3.84	1.4 (max)	10	18	139.5%	207

* WPD: Wilkinson power divider. ^1^ Simulated performance values using a commercial circuit simulator. ^2^ Total electrical length with respect to the center frequency.

## Data Availability

The original contributions presented in the study are included in the article. Further inquiries can be directed to the corresponding author.
